# Scalable Production of Recombinant Membrane Active Peptides and Its Potential as a Complementary Adjunct to Conventional Chemotherapeutics

**DOI:** 10.1371/journal.pone.0139248

**Published:** 2015-09-29

**Authors:** Hussin A. Rothan, Jamunaa Ambikabothy, Ammar Y. Abdulrahman, Hirbod Bahrani, Mojtaba Golpich, Elham Amini, Noorsaadah A. Rahman, Teow Chong Teoh, Zulqarnain Mohamed, Rohana Yusof

**Affiliations:** 1 Department of Molecular Medicine, Faculty of Medicine, University of Malaya, 50603 Kuala Lumpur, Malaysia; 2 Department of Medicine, Faculty of Medicine, Medical Centre of University Kebangsaan Malaysia, 56000 Kuala Lumpur, Malaysia; 3 Department of Chemistry, Faculty of Science, University of Malaya, 50603 Kuala Lumpur, Malaysia; 4 Institute of biological sciences, Faculty of Science, University of Malaya, 50603 Kuala Lumpur, Malaysia; Scuola Internazionale Superiore di Studi Avanzati, ITALY

## Abstract

The production of short anticancer peptides in recombinant form is an alternative method for costly chemical manufacturing. However, the limitations of host toxicity, bioactivity and column purification have impaired production in mass quantities. In this study, short cationic peptides were produced in aggregated inclusion bodies by double fusion with a central protein that has anti-cancer activity. The anticancer peptides Tachiplicin I (TACH) and Latarcin 1 (LATA) were fused with the N- and C-terminus of the MAP30 protein, respectively. We successfully produced the recombinant TACH-MAP30-LATA protein and MAP30 alone in *E*. *coli* that represented 59% and 68% of the inclusion bodies. The purified form of the inclusion bodies was prepared by eliminating host cell proteins through multiple washing steps and semi-solubilization in alkaline buffer. The purified active protein was recovered by inclusive solubilization at pH 12.5 in the presence of 2 M urea and refolded in alkaline buffer containing oxides and reduced glutathione. The peptide-fusion protein showed lower CC_50_ values against cancer cells (HepG2, 0.35±0.1 μM and MCF-7, 0.58±0.1 μM) compared with normal cells (WRL68, 1.83±0.2 μM and ARPE19, 2.5±0.1 μM) with outstanding activity compared with its individual components. The presence of the short peptides facilitated the entry of the peptide fusion protein into cancer cells (1.8 to 2.2-fold) compared with MAP30 alone through direct interaction with the cell membrane. The cancer chemotherapy agent doxorubicin showed higher efficiency and selectivity against cancer cells in combination with the peptide- fusion protein. This study provides new data on the mass production of short anticancer peptides as inclusion bodies in *E*. *coli* by fusion with a central protein that has similar activity. The product was biologically active against cancer cells compared with normal cells and enhanced the activity and selective delivery of an anticancer chemotherapy agent.

## Introduction

Cationic peptides have high aptitudes to interact with cancer cells, especially multidrug resistance (MDR) cells [1,2]. This interaction occurs due to the more negative charge of the membrane of cancer cells compared to normal cells [3]. Recent studies have shown the application of cationic peptides in cancer therapy minimized the side effects of chemotherapy on normal cells [4,5]. The main limitation of developing the cationic peptides for practical applications is the high cost of automated chemical production [3]. For that reason, it is important to develop a cost-effective method for the production of mass quantities of biologically active peptides.

The recombinant forms of anticancer peptides could be considered as an alternative manufacturing strategy. In particular, *Escherichia coli* expression systems have been producing many recombinant proteins in mass quantities with low production costs [6]. However, there are certain limitations to their large-scale production, such as the low efficiency of *E*. *coli* in the formation of disulphide bonds for cysteine-rich peptides [7] and short peptides are almost always produced in soluble, often misfolded forms, which warrant additional steps, as in-column refolding and purification [8].

In this study, the short cationic peptides with anticancer activities were linked to a central protein with similar activity to facilitate production as inclusion bodies in *E*. *coli*. Tachyplesin 1 (TACH) and Latarcin1 (LATA), the cationic peptides with anticancer activities [1,9], were fused to the N- and C-terminus of MAP30 (Momordica protein of 30 kDa), a ribosome-inactivating protein with potential anti cancer activity [10]. Tachyplesin 1, an antimicrobial peptide present in the leukocytes of the horseshoe crab (*Tachypleus tridentatus*), inhibited the proliferation of both cultured tumour and endothelial cells and reduced the colony formation of prostate cancer cells [11,12]. Latarcin1 (LATA) peptide is produced in the venom gland of *Lachesana tarabaevi*, a central Asian spider [13,14]. Recent studies showed a considerable interaction of the LATA peptide with the cell membrane [15]. MAP30 was originally identified as a single chain ribosome inactivating protein. It was isolated from bitter gourd (*Momordica charantia*) seeds, possesses potential anticancer activity against human hepatocellular carcinoma (HepG2) cells *in vitro* and *in vivo* using HepG2-bearing mice models [16]. MAP30 showed anti-tumor effects that attributed to reduce the expression levels of growth factor receptors such as the transmembrane tyrosine kinase receptor HER2 (also known as neu or c-erb-2), which has been implicated in breast cancer [10].

This study provides data on the production of anticancer cationic peptides as a part of a peptide-fusion protein that can be produced by *E*. *coli* and has an effective anticancer function. Our purification strategy depended on the production of the recombinant peptides in inclusion bodies that were easy to isolate, solubilize and refold without column and cleaving steps. The recombinant peptide-fusion protein was produced in a scalable method, exhibited considerable activity against cancer cells compared with normal cells and enhanced the selective delivery of an anticancer chemotherapy agent.

## Methods

### Production of recombinant anti-cancer peptide-fusion protein

#### Chimeric Protein Design

The peptide-fusion protein consisted of TACH and LATA as flanking peptides fused to MAP30 as a central protein. The peptide-fusion protein TACH-MAP30-LATA was constructed by joining the C-terminus of Tach with the N-terminus of MAP30 using a 10 amino-acid linker, and the C-terminus of MAP30 was joined to the N-terminus of LATA using another 10 amino-acid linker.

#### Plasmid construction

The DNA sequence of the recombinant peptide-fusion protein (Tach-MAP30-Lata) was derived from reverse conversion of the amino acid sequence and optimized to *E*. *coli* preferred codons using online software [17,18]. The design lengths of the sense and antisense oligos were 60-mers with 15 bp overlap regions, which were synthesized commercially (1^st^ base, Kuala Lumpur–Malaysia). The Klenow-*Pfu* DNA polymerase method was used to splice and synthesize the entire TACH-MAP30-LATA expression cassette. The TACH-MAP30-LATA expression cassette (and the individual MAP30 gene) was amplified using forward and reverse primers that were designed to include *Bam*HI and *Hind*III restriction sites, respectively. Then, the TACH-MAP30-LATA expression cassette or MAP30 was digested with *Bam*HI and *Hind*III enzymes to facilitate cloning into an appropriate *E*. *coli* expression vector (pTrc-His-A, Invitrogen, Cat. no. V360-20) and transformed into E. coli BL21.

#### Culture Conditions

*Escherichia coli* expression strain BL21 were cultured in LB medium containing 100 μg/mL ampicillin, 1% peptone, 0.5% yeast extract and 0.5% NaCl. A single colony of cells containing fusion plasmid of *Tach-Map30-Lata* or *Map30* inserts was used to inoculate 10 mL of LB broth and grown overnight at 37°C. An overnight culture was used to inoculate one litre of an expression medium. The subculture was grown at 37°C with good aeration (250 rpm shaking) until the OD600 was approximately 0.5. For induction, IPTG was added to the remaining culture to a final concentration of 0.3 mM, and the culture was incubated at 37°C with 250 rpm shaking for 2, 4, 8 and 16 h. At each time point after induction, the quadruplicate of the 1 L culture of recombinant Tach-Map30-Lata or Map30 proteins was analysed for cell mass, inclusion bodies and protein yield; samples were taken for SDS-PAGE and western blot analysis.

#### IB protein separation

The fermentation process of recombinant *E*. *coli* produced the peptide-fusion protein as inclusion bodies. The *E*. *coli* culture was centrifuged at 6000 rpm for 20 min at room temperature. The cell pellet was suspended in lysis buffer (100 mM TrisHCl, 50 mM NaCl, 1 mM of EDTA and 1 mM of PMSF). The cell suspension was sonicated on ice with an amplitude of 10 microns in 15 steps of 30 s alternated with 30 s of rest. The lysate was centrifuged at 10,000 rpm for 15 min to isolate the host cell soluble proteins. The IB pellet was suspended in PBS and incubated with DNase I for 2 h. Then, the pellet was washed three times with IB washing solution (0.5% Triton X-100) followed by two times with deionized water to remove the excess detergent.

#### Solubilization of the purified IBs and protein refolding

The inclusion bodies were isolated and refolded as we described previously [8,19]. In brief, the washed inclusion bodies were suspended with dH2O, pH 12, incubated for 1 h at room temperature and centrifuged at 11,000 rpm for 20 min at 4°C. The pellet was suspended in solubilization buffer containing 100 mM Tris-HCl and 2M urea, pH 12.5, incubated for 1 h at room temperature and centrifuged at 11,000 rpm for 20 min at 4°C. Then, the disulphide bonds were reduced using 5 mM β-mercaptoethanol and incubated at room temperature for 30 min with stirring. Then, ice-cold refolding buffer (100 mM Tris-HCl, 1 mM EDTA, 10% glycerol, 250 mM L-arginine, 1 mM reduced glutathione and 0.5 mM oxidized glutathione [pH 12.0]) was added, and the protein sample was loaded into a dialysis tube. The dialysis was carried out overnight against 200 volumes of 100 mM Tris-HCl, pH 10 to eliminate the residual β-mercaptoethanol and other chemicals.

#### SDS-PAGE and Western Immunoblotting

Purified TACH-MAP30-LATA and MAP30 proteins were first quantified using Bradford method and then separated by SDS-PAGE in reducing and non-reducing conditions (with and without of β-mercaptoethanol). Protein samples were loaded into 12% gels SDS-PAGE and then transferred to a nitrocellulose membrane (1 h, 100 V). Following transfer, the membrane was blocked in Tris-buffered saline with Tween-20 containing 50 g/L skimmed milk for 2 h and then incubated with anti-6XHis tag antibody (Abcam, UK) for 2 h at room temperature. The membrane was washed three times with Tris-buffered saline (15 min each time) and then incubated with mouse anti-IGg antibody conjugated with alkaline phosphatase (Sigma, USA) for 2 h, washed again with Tris-buffered saline as described previously and finally developed with Western Blue® stabilized substrate (Promega, USA).

### Anti-cancer activity

#### Cell Culture

Monolayer breast carcinoma cell lines (MCF-7), Human hepatocellular carcinoma cells (HepG2), arising retinal pigment epithelium (ARPE19) and hepatic human cell line (WRL 68) were obtained from ATCC (USA) and were separately cultured in growth media containing DMEM media, 10% heat inactivated foetal bovine serum (FBS), 100 U/mL penicillin, 100 U/mL streptomycin and 2 mM glutamine. All cell cultures were maintained at standard conditions at 37°C with 5% CO_2_ humidity.

#### Cell Proliferation Assays (MTT Assay)

To evaluate the anti-proliferative activity of the recombinant peptide-fusion protein and the individual antimicrobial peptides, cancer and normal cells were plated onto 96-well plates (1×10^4^ cells per well) in DMEM supplemented with 10% FBS and incubated overnight at 37° and 5% CO. Each peptide was diluted to serial concentrations ranged from 0 to 200 μM (LATA and TACH) and from 0 to 10 μM (MAP30 and Fusion protein) with DMEM media supplemented with 2% FBS. The cell culture was analysed after 72 h in quadruplicate using a commercial non-radioactive cell proliferation assay (Promega, USA) according to the manufacturer’s protocol.

#### Lactate Dehydrogenase Assay (LDH assay)

The effects of the peptides on the cell membrane integrity was investigated by measuring the release of lactate dehydrogenase (LDH) from cancer and normal cells using a Cytotoxicity Detection Kit (Roche Applied Science, Germany) according to the manufacturer’s instructions. Cell lines MCF-7, ARPE19, WRL68 and HepG2 were plated onto 96-well microplates (1×10^4^ cells per well) in DMEM supplemented with 10% FBS and incubated overnight at 37°C and 5% CO_2_. The cells were treated with various concentrations of the peptides (0 to 6 μM) and incubated for another 24 h at 37°C. The extracellular medium from each well was transferred to a new microplate and incubated for 10 min with 100 ml/well reaction mixture, followed by a stop solution. LDH release from cells lysed with 1% Triton X-100 in PBS was defined as 100% leakage and LDH release from untreated cells as 0% leakage. The absorbance at 490 nm was determined using a microplate reader (Tecan Infinite M200 Pro). Dose response curves were presented as the mean value from three independent experiments.

#### ELISA-like cell-based assay

A fluorescence ELISA-like cell-based assay was used to determine the cellular uptake of the peptide-fusion protein as we described previously [20, 21]. Cancer and normal cells were plated onto a black 96-well plate with a transparent bottom and treated with 0 to 2.5 μM of the peptide-fusion protein or MAP30 for 24 h in quadruplicate. Peptide-treated cells were fixed with ice-cold methanol for 15 min at -20°C, washed with PBS and incubated with a coating buffer containing BSA for 1 h at room temperature. The cells were then incubated with mouse anti-6X His tag antibody (Abcam, UK) overnight at 4°C. After the washing steps, the cells were incubated for 30 min with an anti-mouse IgG labelled with a FITC fluorescent dye (Invitrogen, USA). The fluorescence signals were measured using Tecan Infinite M200 Pro fluorescence spectrophotometer (Tecan Group Ltd., Switzerland).

#### Immunostaining

To investigate the cell-penetrating ability and intracellular distribution of the internalized peptides, confocal microscopy was performed on cancer cell lines (MCF-7 and HepG2) and normal cell lines (ARPE19 and WRL68). Briefly, cells (2 × 10^5^) were plated on a glass coverslip placed in a 6-well plate, grown overnight and then incubated with peptide-fusion protein or MAP30 (~1.3 μM for each cell line) for 1 h. The cells were then rinsed three times with phosphate buffered saline (PBS, pH 7.4), fixed with ice-cold methanol for 15 min at -20°C, washed with PBS and incubated with a coating buffer containing BSA for 1 h at room temperature. The cells were then incubated with mouse anti-6X His tag antibody and anti-mouse IgG labelled with FITC as described above. To investigate the morphological changes in the cell membrane, the treated and control HepG2 cells were incubated with CellMask™ Deep Red Plasma membrane Stain (Invitrogen, USA) for 30 min, washed with PBS and examined using confocal microscopy.

#### Selective delivery of an anticancer chemotherapy agent

The selective delivery of doxorubicin, an anticancer chemotherapy agent, by the peptide fusion protein was studied after the treatment of the cells with a combination of a fixed concentration of doxorubicin (2.5 μM) and increased concentrations (0–1.4 μM) of the peptide-fusion protein. The cells were plated onto 96-well microplates (1 × 10^4^ cells per well) in quadruplicate and treated with the drugs. The cell culture was analysed after 72 h using the MTT assay as mentioned above.

## Results

### Production of the Recombinant Anticancer Peptide-fusion protein

**Fig 1:**
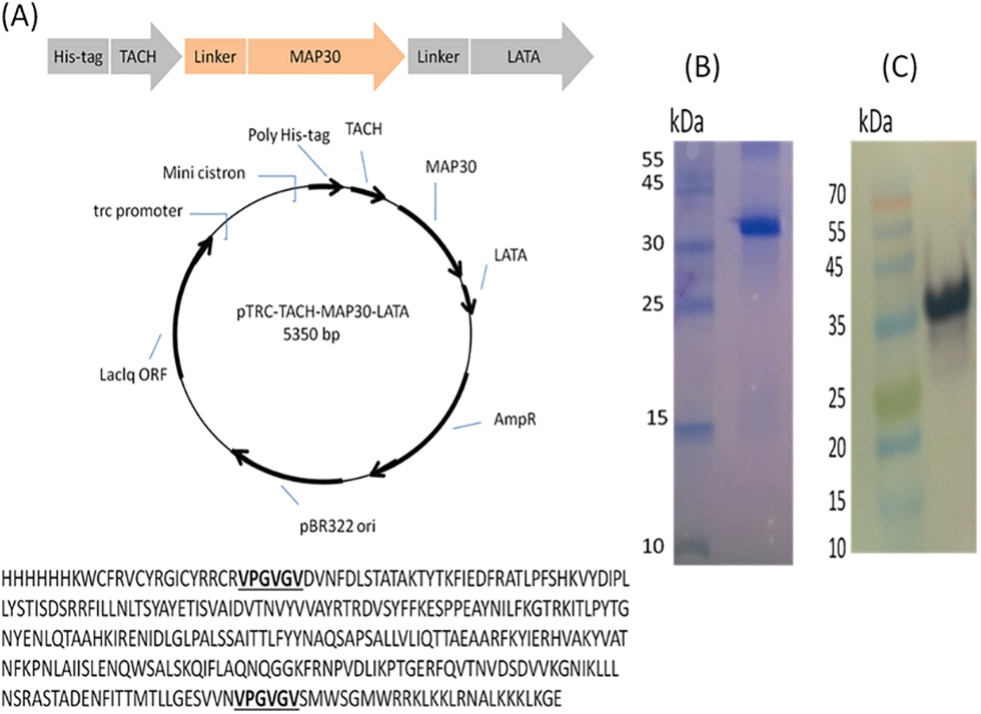
Design and production of the recombinant peptide-fusion protein. **A)** The short peptides TACH and LATA were fused to MAP30 as a central protein using a six amino-acid linker. The TACH-MAP30-LATA expression cassette including 6XHis tag at the N-terminal of fusion protein was cloned into the appropriate *E*. *coli* expression vector and transformed into *E*. *coli* BL21. **B)** SDS-PAGE was done to determine the molecular weight of the purified peptide-fusion protein (~37 kDa). **C)** Immunoblotting using anti-6XHis tag antibody.

This study was initiated by designing the recombinant anticancer peptide- fusion protein *via* joining the TACH peptide to the N-terminal portion of MAP30 and the LATA peptide to the C-terminus using a 6-amino-acid linker on each side (Fig 1A). The molecular weight of the resulting peptide-fusion protein including 6XHis tag (TACH-MAP30-LATA) was approximately 37 kDa (Fig 1B), which was confirmed by immunoblotting using an anti-6XHis tag antibody (Fig 1C).

**Fig 2:**
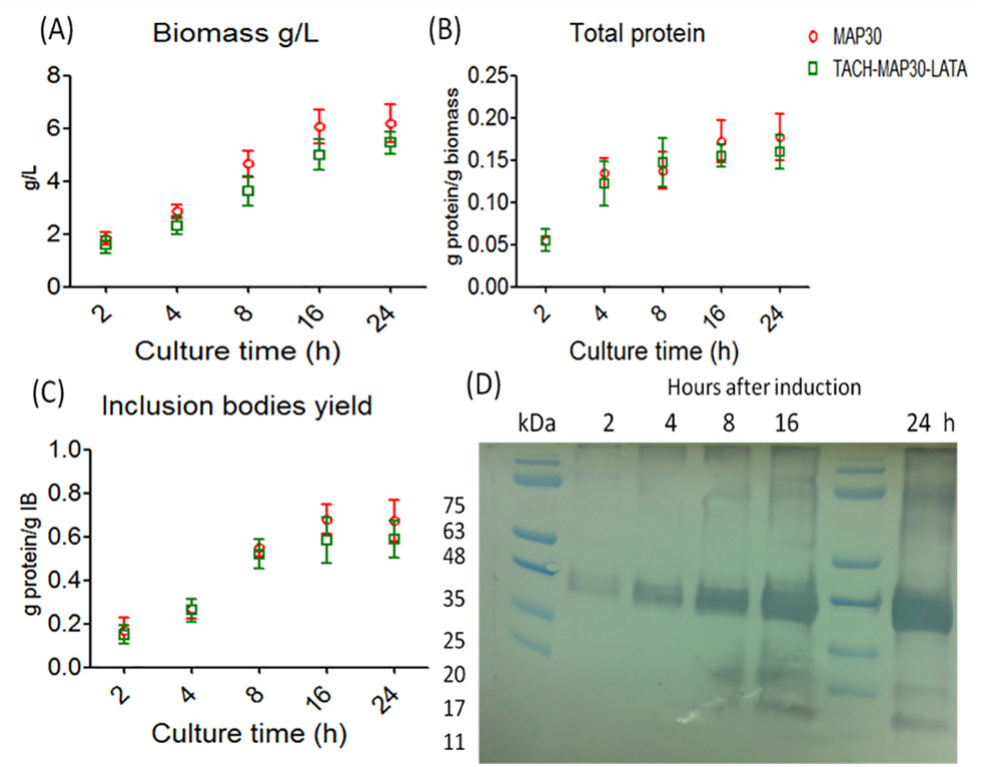
Production of the recombinant peptide-fusion protein (TACH- MAP30-LATA) and MAP30 by a recombinant *E*. *coli*. **A)** Total dried biomass of *E*. *coli* after the chemical induction at all of the time points of the fermentation process. **B)** Relative total recombinant protein to the biomass of TACH-MAP30-LATA and MAP30. **C)** Recombinant protein yield from inclusion bodies. **D)** Western blot analysis of the purified recombinant peptide-fusion protein at each time point of the fermentation process (Two Way-ANOVA, p>0.05).

The peptide-fusion protein was produced insolubly as inclusion bodies as well as MAP30 without fusion for comparison. The results showed the recombinant MAP30 in fusion with TACH and LATA or without fusion was produced at similar levels by *E*. *coli*. The total dried biomass of *E*. *coli* after the chemical induction at all of the time points of the fermentation process was similar (Two Way-ANOVA, p>0.05) for both proteins (after 24 h of incubation, TACH-MAP30-LATA, 5.45±0.42 g/L compared with MAP30, 6.20±0.73 g/L), as presented in Fig 2A. Likewise, the relative total recombinant protein to the biomass of TACH-MAP30-LATA and MAP30 was 0.18±0.03 and 0.16±0.02, respectively (Fig 2B). The results also showed the recombinant protein represented approximately 60% of the inclusion bodies, and no significant difference was observed in this percentage between the peptide-fusion protein (0.59±0.09) and MAP30 (0.68±0.10) as presented in Fig 2C. At each time point of the fermentation process, the purified recombinant peptide-fusion protein was analysed by western blot, which showed increased protein band density at the expected size (~37 kDa) as presented in Fig 2D.

### Solubilization and refolding of the inclusion bodies

**Fig 3:**
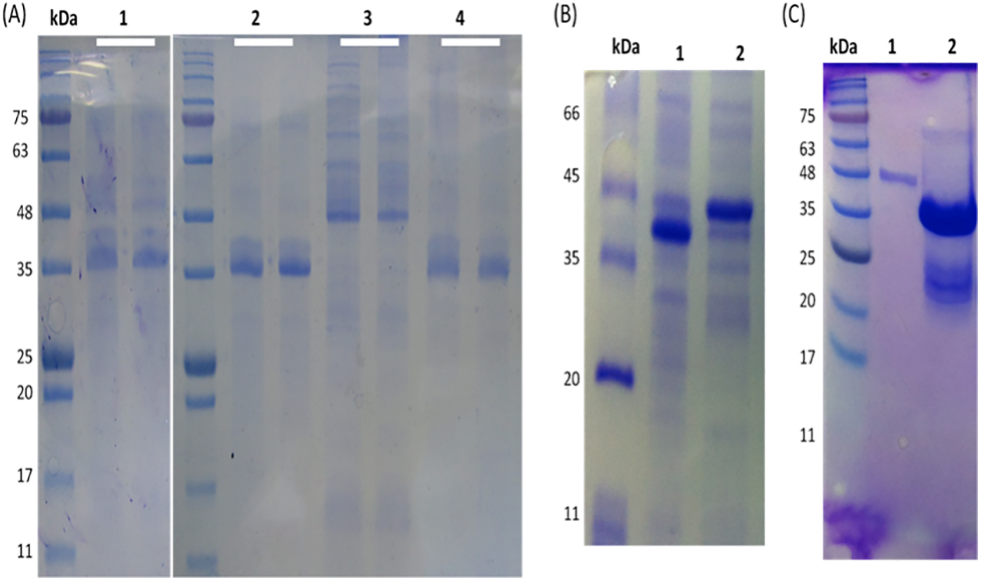
Purification of inclusion bodies by solubilization and refolding in alkaline-based buffer containing redox agents. **A)** Semi-solubilization of inclusion bodies in dH_2_O (pH 12) after 10 min of incubation. L1, the isolated inclusion bodies; L2, purified protein (final product); L3, removing of host cell proteins; L4, remaining insoluble aggregates after final solubilization (each line duplicated). **B)** Refolding of soluble protein in buffer containing oxidized and reduced glutathione to reform the disulphide bridges of TACH. The refolded protein showed different levels under reducing and non-reducing conditions of the SDS-PAGE, indicating the formation of disulphide bridges. **C)** SDS-PAGE analysis of the fusion protein. L1, a single band at the expected size for the monomer. L2, double bands of the fusion protein. The upper faint band at the dimer size and the lower thick band at the monomer size (arrows).

To retrieve the bioactivity of the recombinant peptide-fusion protein, pure inclusion bodies were solubilized and refolded in alkaline-based buffer containing redox agents. Although the inclusion bodies were washed extensively, the host cells contaminants were retained after refolding. Therefore, the purified inclusion bodies were washed with dH_2_O (pH 12) after 10 min of incubation. This step is important to solubilize the host cell proteins that may aggregate with the inclusion bodies; the collected supernatant showed only the host cell contaminants (Fig 3A). The final solubilization of the inclusion bodies was achieved at pH 12.5 in the presence of 2 M urea. The remaining inclusion bodies were insoluble even with higher concentrations of urea, showing that the recombinant peptide-fusion protein was approximately 60% of the total inclusion bodies as shown in Fig 2C and Fig 3A.

The recombinant TACH-MAP30-LATA contains two disulphide bridges in TACH and another in MAP30. Therefore, to refold the soluble protein, the disulphide bridges were reduced by a reducing agent and reformed in the presence of redox agents. SDS-PAGE analysis under reducing and non-reducing conditions showed that the formation of inter-molecular disulphide bonds was more apparent than inta-molecular form even at higher concentrations, which is important for bioactivity (Fig 3B and 3C). The recombinant protein showed considerable antimicrobial activity against gram-positive and gram-negative bacteria (data not shown).

### Anti-Proliferative Activity of the Peptides

**Fig 4:**
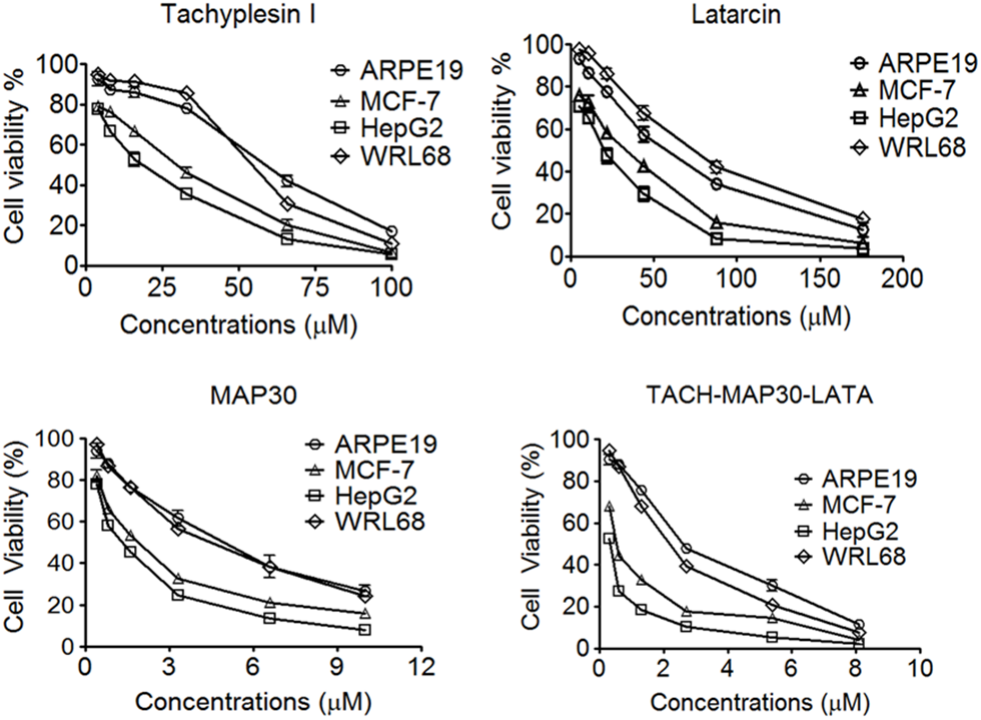
The anti-proliferative activity of the peptides. The individual components of the fusion protein (TACH, LATA and MAP30) showed higher inhibition against cancer cells compared with normal cells in an overall dose range of 0–200 μM based on the maximal toxic concentration of each peptide. The 50% cytotoxic concentration (CC_50_) of the peptide-fusion protein (TACH- MAP30-LATA) towards HepG2 (0.35±0.1 μM) and MCF-7 (0.58±0.1 μM) was significantly lower than WRL68 (1.83±0.2 μM) and ARPE19 (2.5±0.1 μM). Two Way-ANOVA, P<0.001.

The anti-proliferation effect of the peptides was tested using the MTT assay against human cancer cell lines (HepG2 and MCF-7) and normal cell lines (WRL 68 and ARPE19). All of the peptides showed anti-proliferation activities against cancer cells compared with normal cells in a dose-dependent manner. The individual component of the fusion protein (TACH, LATA and MAP30) showed higher inhibition against cancer cells compared with normal cells. Intriguingly, the results showed considerable inhibition of the peptide-fusion protein against cancer cell proliferation compared with the individual peptides. The 50% cytotoxic concentration of the peptide-fusion protein (TACH- MAP30-LATA) towards HepG2 (0.35±0.1 μM) and MCF-7 (0.58±0.1 μM) was significantly (Two Way-ANOVA, P<0.001) lower than WRL68 (1.83±0.2 μM) and ARPE19 (2.5±0.1 μM) as presented in Fig 4.

### Characterization of the Peptide-fusion protein Uptake by Cancer and Normal cells

**Fig 5:**
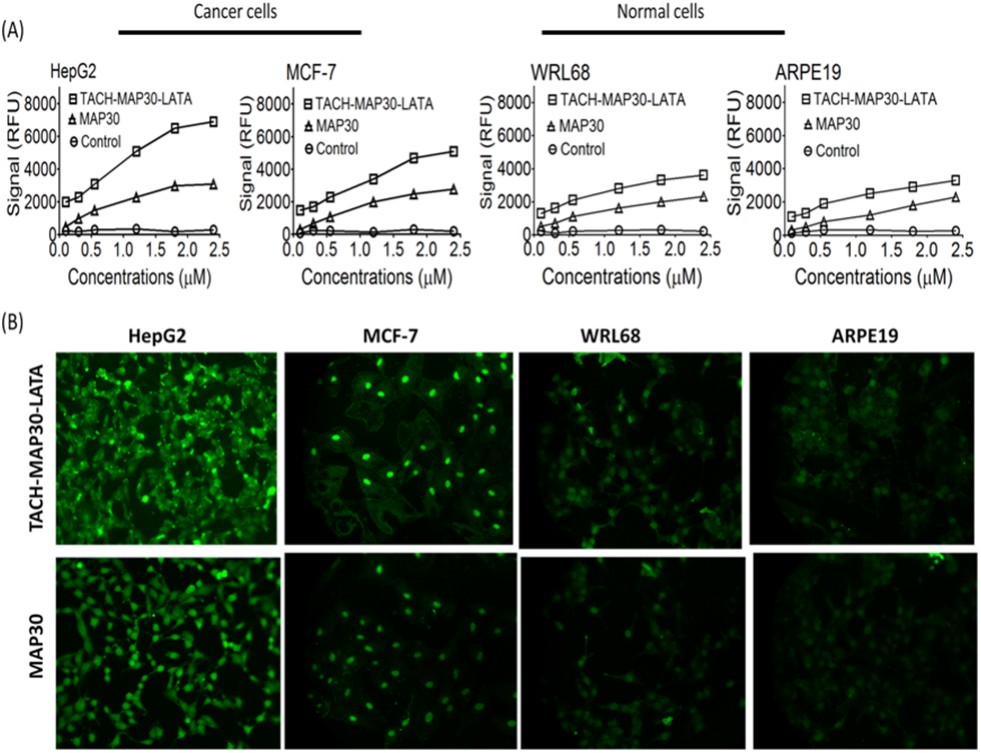
Cellular uptake of the peptide-fusion protein (TACH-MAP30-LATA) and MAP30 by cancer and normal cells. **A)** Fluorescence ELISA-like cell-based assay at 0 to 2.5 μM showed the peptide-fusion protein was internalized more efficiently than MAP30 into cancer cells (HepG2 and MCF-7) compared with normal cells (WRL68 and ARPE19) in a dose-dependent manner**. B)** Confocal laser microscopy analysis showed TACH-MAP30-LATA was preferentially uptaken by HepG2 and MCF-7, compared with normal cells (WRL68 and ARPE19), whereas the cellular uptake of MAP30 was less in all cell lines.

To compare the quantity of the internalized peptide-fusion protein in different cell types, we performed a Fluorescence ELISA-like cell-based assay. The peptide-fusion protein was internalized more efficiently than MAP30 into cancer cells (HepG2 and MCF-7) compared with normal cells (WRL68 and ARPE19) in dose- dependent manner. The HepG2-treated cells showed higher cellular uptake of the peptide-fusion protein (approximately 2.2-fold at 2.5 μM) than MAP30. The cellular uptake of the peptide-fusion protein (TACH-MAP30-LATA) at 2.5 μM by MCF-7, WRL68 and ARPE19 was 1.8-fold, 1.5-fold and 1.4-fold compared with MAP30 (Fig 5A). This finding indicates that the fusion of the short cationic peptides facilitated the internalization of MAP30 into the target cells. Further investigation was conducted using confocal laser microscopy. TACH-MAP30-LATA was preferentially uptaken by HepG2 and MCF-7 compared with normal cells (WRL68 and ARPE19), whereas the cellular uptake of MAP30 was less in all cell lines (Fig 5B). These results clearly show that the peptide-fusion protein has cancer cell specificity higher than MAP30 without the fusion peptides.

### Cell membrane integrity

**Fig 6:**
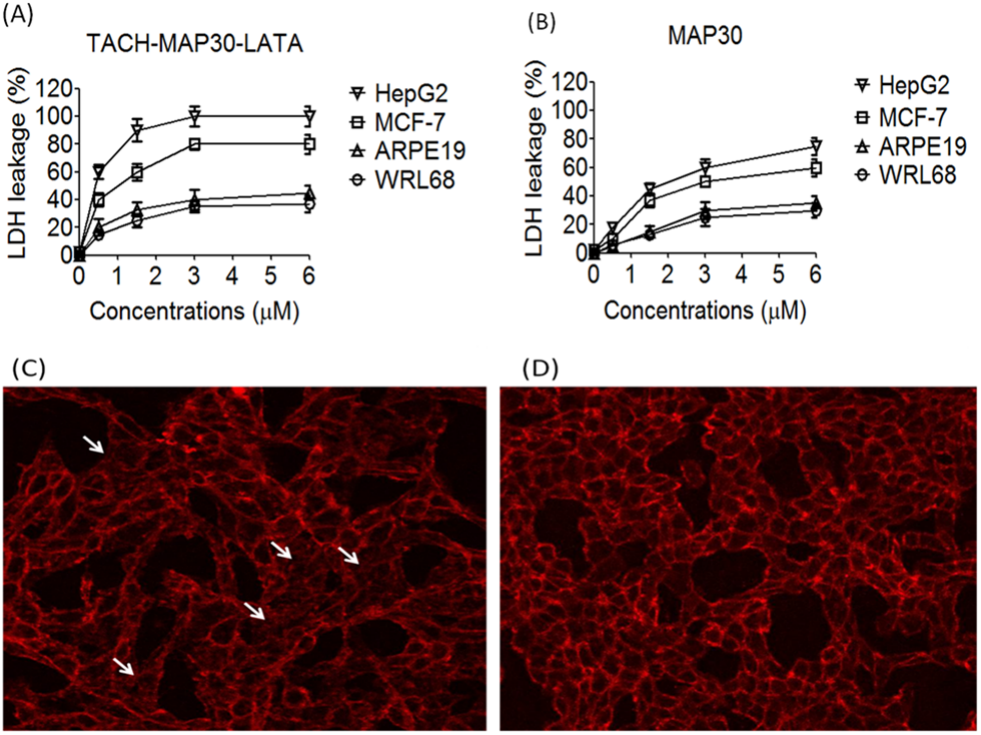
Cell membrane integrity after the treatment with the peptides-fusion protein. **A)** TACH-MAP30-LATA induced significant LDH leakage from both cancer (approximately 80–100%) and normal cell lines (approximately 40%) at 3 μM. **B)** MAP30-treated cancer cells showed less than 75% of LDH leakage at 6 μM, while the normal cells showed less than 35% **C)** Treatment of HepG2 cells with the peptide-fusion protein, the cells showed apparent plasma membrane damage and the cellular shape was irregular with disappeared borders (arrows) compared with untreated cells **(D)** Untreated HepG2 cells (Two Way ANOVA, P<0.001).

To assess the cell membrane integrity after the treatment with the peptides-fusion protein, the LDH leakage was measured in cancer cell lines (HepG2 and MCF-7) and normal cell lines (WRL68 and ARPE19). Peptide-fusion protein treatment (0–6 μM) was associated with LDH leakage from cancer cells; the leakage gradually increased with TACH-MAP30-LATA concentrations, reaching approximately 80–100% at 6 μM. However, normal cells showed less than 45% of LDH leakage at similar concentrations. TACH-MAP30-LATA induced significant (Two Way ANOVA, P<0.001) LDH leakage from both cancer (approximately 80–100%) and normal cell lines (approximately 40%) at 3 μM (Fig 6A). MAP30-treated cancer cells showed less than 75% of LDH leakage at 6 μM, while the normal cells showed less than 35% (Fig 6B). In addition, no haemolysis was induced after the erythrocytes were treated with 6 μM of MAP30-LATA or MAP30 (data not shown).

This result was further analysed by confocal laser microscopy using deep plasma membrane stain. After the treatment of HepG2 cells with the peptide-fusion protein, the cells showed apparent plasma membrane damage, and the cellular shape was irregular with disappeared borders compared with untreated cells (Fig 6C and 6D).

### Co-treatment of the Peptide-fusion protein and Doxorubicin selectively inhibits cancer cell growth in vitro

**Fig 7:**
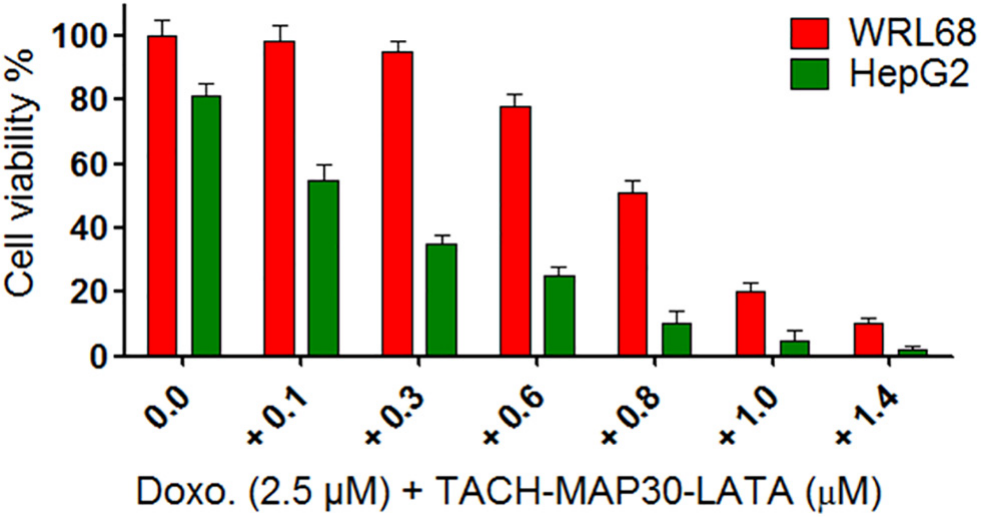
Treatment of liver normal cells (WRL68) and liver cancer cells (HepG2) with combinations of doxorubicin and increasing concentrations of the peptide-fusion protein. The dose of doxorubicin (2.5 μM) that showed 100% of normal cell viability and approximately 80% of cancer cells viability was used with increasing concentrations (0–1.4 μM) of TACH-MAP30-LATA. The results showed significant reduction in cancer cell viability compared with normal cells (Two Way ANOVA, P<0.001).

We next determined whether the peptide-fusion protein was able to enhance the selectivity and efficacy of an anticancer chemotherapy agent. Combinations of doxorubicin, an anticancer chemotherapy agent, with increased concentrations of the peptide-fusion protein were applied on liver normal cells (WRL68) and liver cancer cells (HepG2). The dose of doxorubicin (2.5 μM) that showed 100% of normal cell viability and approximately 80% of cancer cells viability was used with increasing concentrations (0–1.4 μM) of TACH-MAP30-LATA. The results showed significant (Two Way ANOVA, P<0.001) reduction in cancer cell viability compared with normal cells. The considerable reduction in cancer cell viability with a lesser effect on normal cell viability was observed at 0.1–0.6 μM in combination with 2.5 μM of doxorubicin (Fig 7). Our results showed a more considerable interaction of the peptide-fusion protein (TACH-MAP30-LATA) with the cell membrane of cancer cells than normal cells. Consequently, this finding is significant enough to pave the way for a cancer chemotherapy administration regimen that is more selective and effective.

## Discussion

Here, we reported the scalable production of active recombinant anticancer peptides in *E*. *coli via* fusion with a central protein that has similar activity. The growing interest with anticancer peptides is that they possess high specificity and selectivity in their interactions, and this ultimately reduces the possible side effects and maximizes the potencies of action [22]. The recombinant forms of these peptides have been considered as an alternative to the high cost production *via* chemical synthesis. However, the roadblock of the scalable production of soluble recombinant peptides is the column purification [23]. Our purification strategy was optimized for production of the peptide-fusion protein (TACH-MAP30-LATA) as insoluble inclusion bodies in *E*. *coli*. The expression levels of MAP30 and TACH-MAP30-LATA were approximately similar despite the extra sequences of TACH and LATA that are rich in arginine and lysine residues.

In many cases, the recombinant protein accumulates as insoluble inactive aggregates known as inclusion bodies. The inclusion bodies can be an advantage for large scale production because they are easily isolated by multiple washing steps and centrifugation to yield highly concentrated and relatively pure protein [24]. Moreover, inclusion body formation protects the protein from the proteolytic activity of the host cell enzymes [25]. In addition, the toxic proteins or antimicrobial peptides may not inhibit host cell growth when present in the inactive form as inclusion bodies [26]. In this study, the anticancer activity of the peptide-fusion protein was retrieved after solubilization of the inclusion bodies in the presence of a denaturing agent followed by refolding of the soluble protein in alkaline buffer containing redox agents. Although many studies on refolding technology exist in the literature, the methods optimized for chimeric proteins such as our peptide-fusion protein have been mostly limited. In this context, the refolding strategy may extend to the stage of chimeric protein design. The presence of a six amino acid flexible liker among the three units of the peptide-fusion protein may be necessary for each unit to refold autonomously. Furthermore, the linker after TACH provides enough space for the disulphide bridge formation. The biological activity of the peptides largely depends on their secondary structure, which is maintained by the formation of intra-molecule disulphide bonds [27]. Therefore, reformation of the disulphide bonds was achieved in this study by reducing the existence bonds and reforming them in the presence of reduced and oxidized glutathione as previously reported [28]. Then, the biologically active peptide-fusion protein showed considerable anticancer activity against cancer cells compared with normal cells.

In general, cancer is characterized by uncontrolled cellular growth and the spread of abnormal cells, which leads to death [29]. Although there has been advanced progress in cancer treatment, cancer still remains a major cause of morbidity and mortality worldwide [1]. The five year worldwide prevalence of cancer is estimated to be nearly 29 million people [30]. Among different treatments, chemotherapy is commonly used to kill cancer cells. However, chemotherapy treatment usually leads to killing rapidly dividing normal cells, such as those in the bone marrow (causing myelosuppression), epithelial cells lining the gastrointestinal tract and hair follicles [31,32]. Therefore, chemotherapy treatment causes cytotoxic effects on normal cells leading to negative effects on patient compliance. Furthermore, chemotherapy treatment has been shown to have insignificant efficiency against the growth of multidrug resistance (MDR) neoplasic cells.

Doxorubicin is a chemotherapy drug, commonly used in the treatment of a wide range of cancers, including haematological malignancies (blood cancers, such as leukaemia and lymphoma), many types of carcinoma (solid tumours) and soft tissue sarcomas [33]. In our study, the results showed high selectivity and efficiency of the doxorubicin/peptide-fusion protein combination against cancer cells. This finding is important in the treatment of MDR cells because they have been known to be a consequence of the membrane acting mechanism, especially P-glycoprotein [34,35]. This protein serves as an efflux pump in the cell membrane for various anticancer agents. It has been shown that MDR cells are usually associated with overexpression of P-glycoprotein, which proceeds to pump anticancer drugs outside the cell [36]. In addition, cancer cells also frequently become resistant to chemotherapy as a result of a change in cellular components, including elevated drug-detoxifying enzyme expression and drug transporters, altered interactions between the drug and cellular target, an increased ability to repair DNA damage and defects in the cellular machinery that mediate apoptosis [37]. To that end, the discovery of novel and potent anticancer agents that act by different mechanisms warrants urgency [38]. Therefore, in our study, the membrane active peptides might be facilitating the cellular uptake of doxorubicin in cancer cells compared with normal cells. However, further studies are still required to explain the actual interaction of the peptide-fusion protein with doxorubicin.

In conclusion, we report in this study that the membrane active peptides can be produced on large scale as a recombinant peptide-fusion protein in *E*. *coli*. The peptide-fusion protein is more biologically active against cancer cells than normal cells. It could also enhance the selective delivery of an anticancer chemotherapy agent.
